# Caspase-2 is involved in cell death induction by taxanes in breast cancer cells

**DOI:** 10.1186/1475-2867-13-42

**Published:** 2013-05-15

**Authors:** Michael Jelínek, Kamila Balušíková, Dana Kopperová, Vlasta Němcová-Fürstová, Jan Šrámek, Julie Fidlerová, Ilaria Zanardi, Iwao Ojima, Jan Kovář

**Affiliations:** 1 Department of Cell and Molecular Biology, Third Faculty of Medicine, Charles University, Ruská 87, 100 00, Prague 10, Czech Republic; 2 Institute of Chemical Biology and Drug Discovery, State University of New York at Stony Brook, Stony Brook, NY, USA

**Keywords:** Caspase-2, Cell death, Taxanes, Breast cancer cells

## Abstract

**Background:**

We studied the role of caspase-2 in apoptosis induction by taxanes (paclitaxel, novel taxane SB-T-1216) in breast cancer cells using SK-BR-3 (nonfunctional p53, functional caspase-3) and MCF-7 (functional p53, nonfunctional caspase-3) cell lines.

**Results:**

Both taxanes induced apoptosis in SK-BR-3 as well as MCF-7 cells. Caspase-2 activity in SK-BR-3 cells increased approximately 15-fold within 48 h after the application of both taxanes at the death-inducing concentration (100 nM). In MCF-7 cells, caspase-2 activity increased approximately 11-fold within 60 h after the application of taxanes (300 nM). Caspase-2 activation was confirmed by decreasing levels of procaspase-2, increasing levels of cleaved caspase-2 and the cleavage of caspase-2 substrate golgin-160. The inhibition of caspase-2 expression using siRNA increased the number of surviving cells more than 2-fold in MCF-7 cells, and at least 4-fold in SK-BR-3 cells, 96 h after the application of death-inducing concentration of taxanes. The inhibition of caspase-2 expression also resulted in decreased cleavage of initiator caspases (caspase-8, caspase-9) as well as executioner caspases (caspase-3, caspase-7) in both cell lines after the application of taxanes. In control cells, caspase-2 seemed to be mainly localized in the nucleus. After the application of taxanes, it was released from the nucleus to the cytosol, due to the long-term disintegration of the nuclear envelope, in both cell lines. Taxane application led to some formation of PIDDosome complex in both cell lines within 24 h after the application. After taxane application, p21^WAF1/CIP1^ expression was only induced in MCF-7 cells with functional p53. However, taxane application did not result in a significant increase of PIDD expression in either SK-BR-3 or MCF-7 cells. The inhibition of RAIDD expression using siRNA did not affect the number of surviving SK-BR-3 and MCF-7 cells after taxane application at all.

**Conclusion:**

Caspase-2 is required, at least partially, for apoptosis induction by taxanes in tested breast cancer cells. We suggest that caspase-2 plays the role of an apical caspase in these cells. Caspase-2 seems to be activated via other mechanism than PIDDosome formation. It follows the release of caspase-2 from the nucleus to the cytosol.

## Background

Taxanes represent a well-known but relatively new group of anticancer drugs. There are two established (classical) taxanes, paclitaxel (Taxol®) and docetaxel (Taxotere®), currently used for treatment of breast and ovarian cancer as well as head and neck, lung and prostate cancer [1,2]. In addition to the aforementioned classical taxanes, novel taxanes have been developed. They represent a new generation of taxoids (taxane analogs). They are not yet used in clinical practice but they are substantially more effective in resistant cancer cells *in vitro* and *in vivo*[3-7].

Taxanes are mitotic poisons. They bind to the β subunit of the tubulin heterodimer, thereby stabilizing microtubules and inhibiting their depolymerization [8-10]. In this way, taxanes are thought to block progression through the M-phase of the cell cycle [9,11]. However, the relationship between mitotic arrest and the induction of cell death by taxanes remains unclear [12-14].

The molecular mechanism of cell death induction by taxanes is not fully understood either. It has been previously shown that apoptosis induced by taxanes seems to be p53 independent [4,15]. On the other hand, several findings concerning cytochrome c release, caspase-9 activation and caspase-3 activation strongly indicate, that at least in some cases, a mitochondrial pathway is involved in apoptosis induction by taxanes [4,16,17]. However, alternative nonmitochondrial pathways could also be involved [4,7,18], including caspase-8 activation [7,14,19]. Recently, the role of caspase-2 in apoptosis induction by taxanes has come under consideration [7,14,20,21].

Caspase-2 is ubiquitously expressed and represents an evolutionarily highly conserved mammalian caspase. However, its precise physiological function has not been identified. Several lines of evidence point to caspase-2 as a major player in apoptosis induction [22,23]. Procaspase-2 interacts with other proteins, such as CARD-containing RAIDD protein, via its caspase recruitment domain (CARD). RAIDD interacts with another death domain containing protein PIDD *via* its death domain [24]. The complex of procaspase-2, RAIDD and PIDD, known as PIDDosome, facilitates caspase-2 activation. PIDD is a p53-inducible protein [23,25]. In some cases, PIDD seems to function as a regulator of caspase-2 activity [26]. However, caspase-2 activation independent of p53, as well as RAIDD and PIDD, has also been reported, e.g. in cases of cell death via a mitotic catastrophe [27-30]. Caspase-2 has been found in the cytosol, Golgi complex and mitochondria. It is also present in the nucleus. Active caspase-2 specifically cleaves golgin-160 which is present in the Golgi complex [31].

It has been suggested that caspase-2 functions as the most apical caspase when apoptosis is induced by DNA damage and cytotoxic stress [32,33]. The involvement of caspase-2 activation in apoptosis of breast cancer cells, induced by various stimuli, has also been found [27,34-36]. Several other studies have also demonstrated caspase-2 activation in various types of cancer cells following apoptosis induction by taxanes [21,37,38].

We have previously found that caspase-2 is significantly activated in breast cancer cells (together with the activation of caspase-3, caspase-9 and caspase-8) following apoptosis induction by taxanes [7,14]. We have also shown that the mitochondrial pathway is not, at least in some cases, the predominant pathway of apoptosis induction by taxanes in breast cancer cells, and that caspase-2 may be a major player in this process [7]. In our present study, we investigated the role of caspase-2 in apoptosis induction by taxanes in breast cancer cells. We used breast cancer cells SK-BR-3 (nonfunctional p53, functional caspase-3) and MCF-7 (functional p53, nonfunctional caspase-3) as an experimental model and tested both classical (paclitaxel) and novel (SB-T-1216) taxanes. We demonstrated that caspase-2 is required for apoptosis induction by taxanes in the tested breast cancer cells, probably as an apical caspase. Caspase-2 is activated via other mechanism than PIDDosome formation.

## Results

### Effect of taxanes on growth and survival

**Figure 1:**
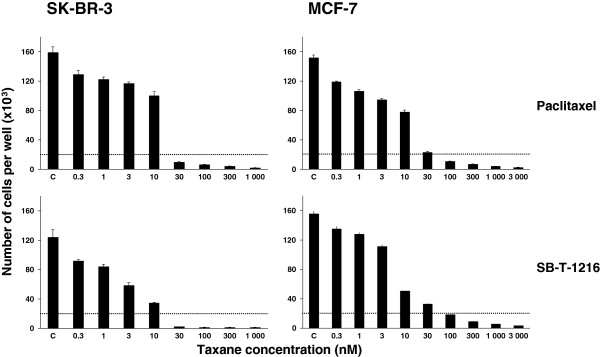
**Effect of paclitaxel and SB-T-1216 (0.3-3000 nM) on the growth and survival of SK-BR-3 and MCF-7 cells. **Control cells (C) were incubated without taxane. The cells were seeded at 20×103 cells/100 µl of medium per well. The number of cells of the inoculum is shown as a dotted line. The number of living cells was determined after 96 h of incubation (see “Materials and Methods“). Each column represents the mean of 8 separate cultures ± SEM.

The effects of paclitaxel and SB-T-1216 on growth and survival of SK-BR-3 cells were tested over a wide range of concentrations (0.3-1000 nM). Paclitaxel and SB-T-1216 both induced death of SK-BR-3 cells within 96 h of incubation at a concentration of 30 nM and higher concentrations. The C_50_ values (concentration of taxanes resulting in 50% living cells compared to controls after 96 h of incubation) were 15 nM and 3 nM for paclitaxel and SB-T-1216, respectively (Figure 1).

In the case of MCF-7, the effects of taxanes were also tested over a wide range of concentrations (0.3-3000 nM). Both paclitaxel and SB-T-1216 induced the death of MCF-7 cells within 96 h of incubation at a concentration of 100 nM and higher concentrations. The C_50_ values of paclitaxel and SB-T-1216 were similar, 5 nM and 8 nM, respectively (Figure 1).

The data showed that MCF-7 cells were more resistant to cell death induction by both taxanes at higher concentrations (30 nM and higher concentrations) than SK-BR-3 cells. In MCF-7 cells, paclitaxel and SB-T-1216 exerted similar effects. However, in SK-BR-3 cells, SB-T-1216 seemed to be more efficient than paclitaxel (Figure 1).

On the basis of our data, we selected 100 nM and 300 nM as the cell death-inducing concentrations, i.e. the lowest concentration having complete death-inducing effect, of tested taxanes for SK-BR-3 cells and MCF-7 cells, respectively. These concentrations were used in subsequent experiments.

### Effect of taxanes on caspase-2 activity

Employing a commercial kit and flow cytometry (see “Materials and Methods”), we tested the time course of caspase-2 activation in both SK-BR-3 and MCF-7 cells after taxane application.

**Figure 2:**
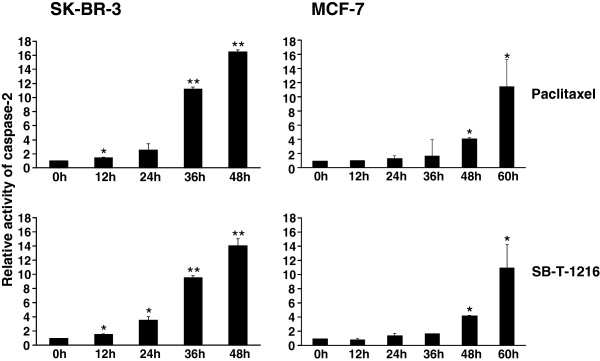
**Effect of paclitaxel and SB-T-1216 on the activity of caspase-2 in SK-BR-3 and MCF-7 cells.** After 0, 12, 24, 36, 48 and 60 h of incubation with paclitaxel or SB-T-1216 (100 nM for SK-BR-3 cells and 300 nM for MCF-7 cells), the activity of caspase-2 was measured by flow cytometry employing CaspGLOWTM Active Caspase Staining Kit (see “Materials and Methods“). Mean fluorescence at 0 h was 23.9 for SK-BR-3 cells and 28.5 for MCF-7 cells. Each column represents the mean of 2 experimental values ± SEM. *P < 0.05, **P < 0.01 when comparing the effect with that at 0 h.

Caspase-2 activity in SK-BR-3 cells increased within 48 h after the application of paclitaxel and SB-T-1216 at death-inducing concentrations (100 nM) approximately 16-fold and 14-fold, respectively. A substantial increase (approximately 10-fold for both taxanes) was seen 36 h after taxane application as well as a noticeable increase (about 3-fold) 24 h after application (Figure 2).

In the case of MCF-7 cells, caspase-2 activity increased within 60 h after the application of both taxanes at death-inducing concentrations (300 nM) approximately 11-fold. A significant increase (approximately 4-fold for both taxanes) was seen 48 h after taxane application and a noticeable increase (about 2-fold) 36 h after application (Figure 2).

**Figure 3:**
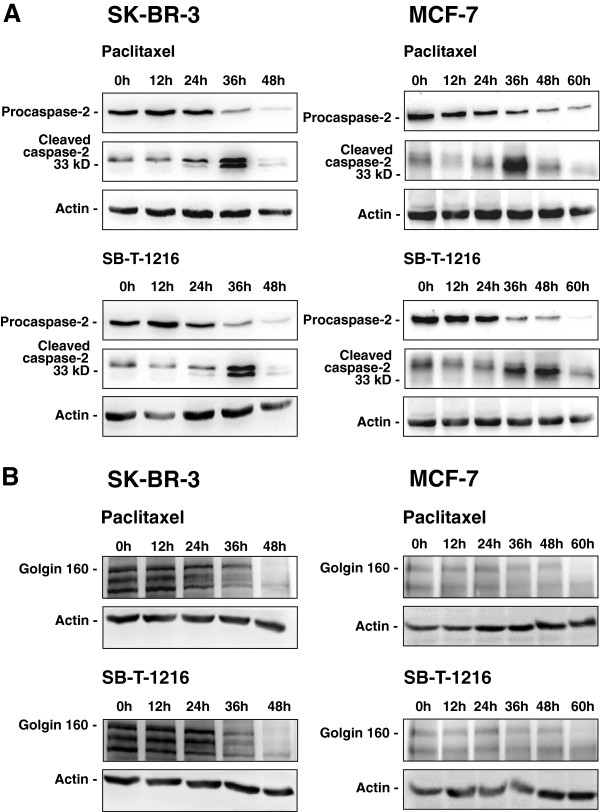
**Effect of paclitaxel and SB-T-1216 on (A) the level of procaspase-2, cleaved caspase-2, and (B) the level of golgin-160 in SK-BR-3 and MCF-7 cells.** After 0, 12, 24, 36, 48 and 60 h of incubation with paclitaxel or SB-T-1216 (100 nM for SK-BR-3 cells and 300 nM for MCF-7 cells), levels of procaspase-2, cleaved caspase-2 and levels of golgin-160 were determined using western blot analysis and relevant antibodies (see “Materials and Methods”). Actin levels were used to confirm equal protein loading. The data shown were obtained in one representative experiment of three (procaspase-2, cleaved caspase-2) or two (golgin 160) independent experiments. MCF-7 data demonstrating the level of procaspase-2 and the level of cleaved caspase-2 are from different experiments.

In order to confirm caspase-2 activation, we assessed the cleavage of procaspase-2 by measuring the level of procaspase-2 using western blot analysis. Procaspase-2 level decreased significantly 36 h after the application of both taxanes in SK-BR-3 cells. After 48 h, the level of procaspase-2 in SK-BR-3 cells was very low (Figure 3A). Concerning MCF-7 cells, some decrease in procaspase-2 level was seen 24 h after taxane application, but a more significant decrease was seen 36 h and 48 h after application. After 60 h, the level of procaspase-2 in MCF-7 cells was very low (Figure 3A).

Decreasing procaspase-2 level points at procaspase-2 cleavage resulting from formation of active caspase-2. The decrease of procaspase-2 level in SK-BR-3 cells correlated with increased level of the cleaved form 36 h after taxane application. However, 48 h after taxane application cleaved caspase-2 disappeared (Figure 3A). Similarly, the decrease of procaspase-2 level in MCF-7 cells correlated with increased level of the cleaved form 36 h and 48 h after taxane application. Again, 60 h after taxane application we can see decreasing level of cleaved caspase-2 (Figure 3A).

The levels of caspase-2 substrate golgin-160 using western blot analysis were also assessed. A significant decrease in golgin-160 level corresponded with decreased procaspase-2 level in both SK-BR-3 and MCF-7 cells after application of both taxanes (Figure 3A,B).

### Effect of the inhibition of caspase-2 expression on taxane induced cell death

Employing RNA interference (see “Materials and Methods”), we assessed the effect of specific inhibition of caspase-2 expression on cell death induction after taxane application.

**Figure 4:**
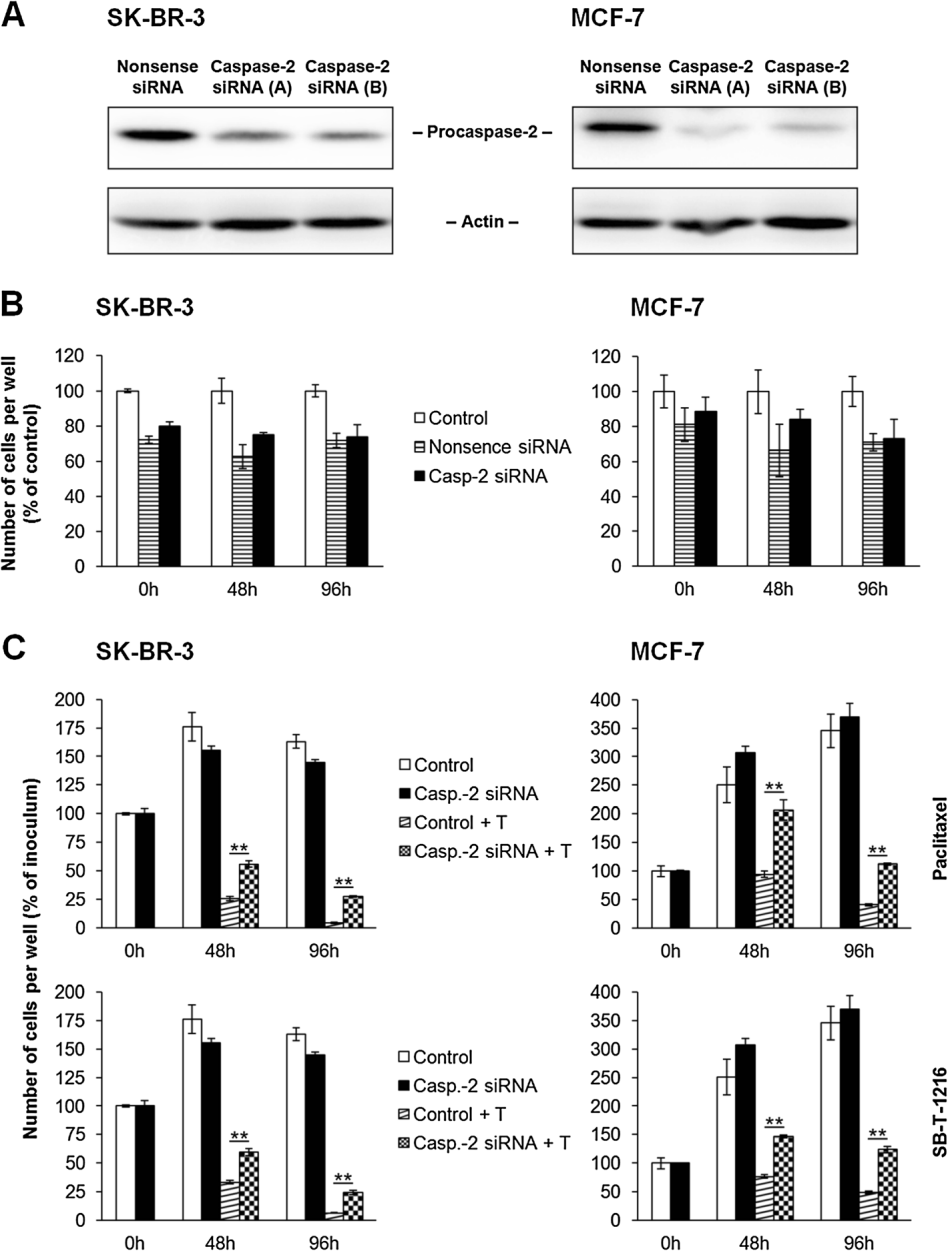
**Effect of the inhibition of caspase-2 expression on the growth and survival of SK-BR-3 and MCF-7 cells after paclitaxel and SB-T-1216 treatment.** (**A**) Efficiency of caspase-2 suppression by two employed specific siRNAs, i.e. **A** and **B**, in SK-BR-3 and MCF-7 cells is shown. Levels of procaspase-2 were determined using western blot analysis and relevant antibodies (see “Materials and Methods”). Actin levels were used to confirm equal protein loading. (**B**) Effect of nonsense siRNA and specific caspase-2 siRNA on the growth and survival of SK-BR-3 and MCF-7 cells without taxane treatment is also shown. (**C**) The effect of specific caspase-2 siRNA on the growth and survival of SK-BR-3 and MCF-7 cells after taxane (T) treatment (100 nM for SK-BR-3 cells and 300 nM for MCF-7 cells) is presented. The cells were seeded at 7 × 10^3^ cells/200 μl of medium per well and prepared as described (see “Materials and Methods”). After 0, 48 and 96 h of incubation, the number of living cells was determined (see “Materials and Methods”). Each column represents the mean of 4 separate cultures ± SEM. *P < 0.05, **P < 0.01 when comparing the effect.

First, the efficiency of the RNA interference was tested. The inhibition of caspase-2 expression was about 80% compared to control (Figure 4A) in SK-BR-3 cells and more than 80% in MCF-7 cells (Figure 4A). Moreover, nonsense siRNA or specific caspase-2 siRNA did not significantly affect cell growth or survival in either cell line. However, cells transfected with siRNAs seemed to grow slightly slowlier in comparison with control (Figure 4B).

After 48 h of incubation with taxanes at death-inducing concentrations (100 nM), the inhibition of caspase-2 expression resulted in an approximately 2-fold increase in the number of surviving SK-BR-3 cells. It represents a statistically significant increase from 26% to 60% of the original number of cells for paclitaxel and from 33% to 64% for SB-T-1216. After 96 h, the effect was even more pronounced. In the case of paclitaxel, the number of surviving SK-BR-3 cells increased from 4% to 30% and from 7% to 26% for SB-T-1216 (Figure 4C).

As for MCF-7 cells, the inhibition of caspase-2 expression, after the application of both paclitaxel and SB-T-1216 at death-inducing concentrations (300 nM), increased the number of surviving cells roughly 2-fold after 48 h and 96 h of incubation. After 48 h of incubation, there was a statistically significant increase from 96% to 203% of the original number of cells for paclitaxel and from 78% to 147% for SB-T-1216. After 96 h, the number of surviving cells increased from 42% to 112% and from 50% to 121% for paclitaxel and SB-T-1216, respectively (Figure 4C). It was also a statistically significant increase. These data demonstrate that MCF-7 cells grew even after application of both taxanes at death-inducing concentrations when caspase-2 expression was inhibited (Figure 4C).

### Effect of the inhibition of caspase-2 expression on taxane induced activation of caspase-8, -9, -3 and -7

Using siRNA technique (see “Materials and Methods”), we also assessed the effect of specific inhibition of caspase-2 expression on the activation of caspase-8, -9,-3 and -7 after taxane application.

**Figure 5:**
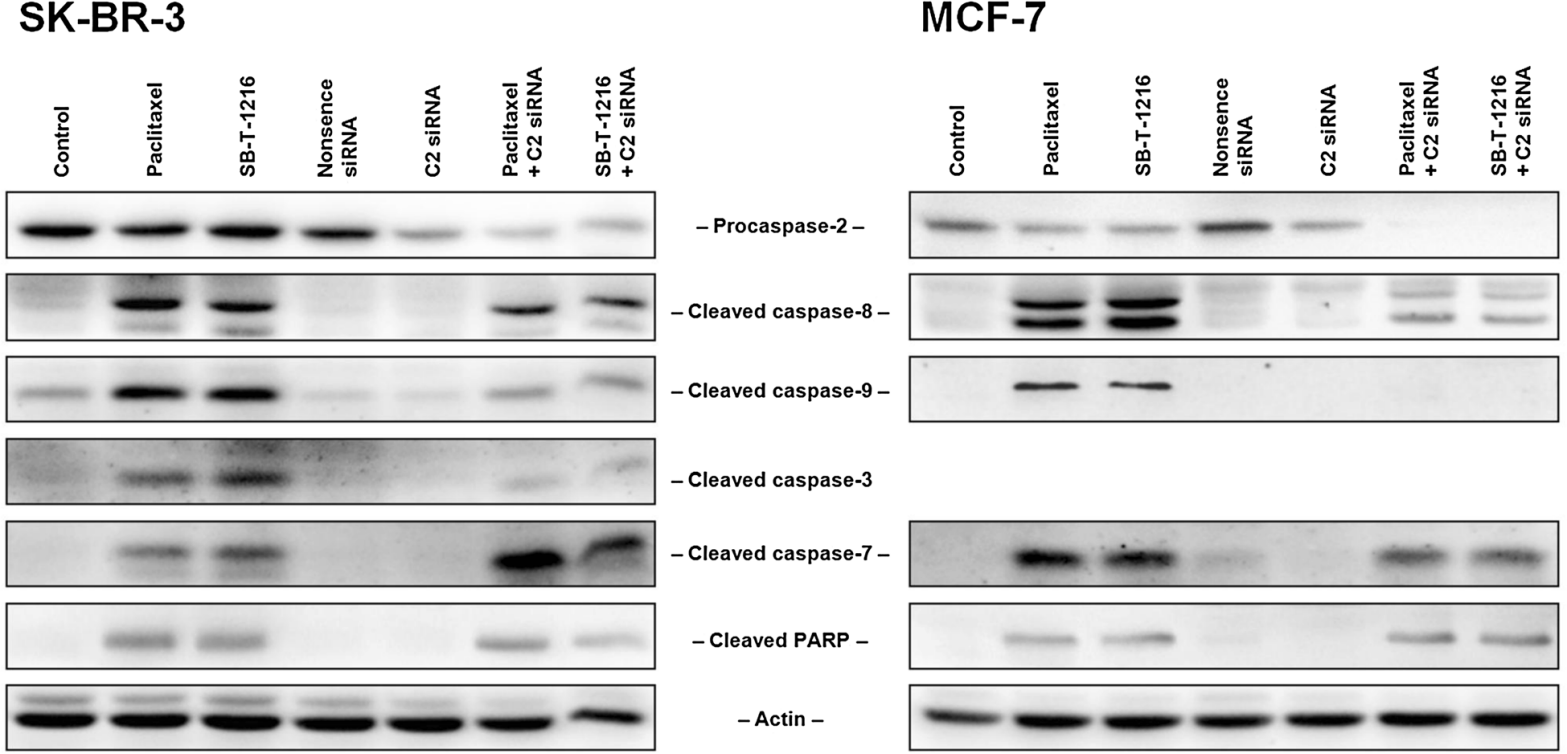
**Effect of the inhibition of caspase-2 expression on the activation of caspase-8, -9, -3 and -7 in SK-BR-3 and MCF-7 cells after paclitaxel and SB-T-1216 treatment.** A specific caspase-2 siRNA (C2 siRNA) was used. Control cells were incubated without taxane and siRNAs. Effects of nonsense siRNA as well as caspase-2 siRNA without taxane treatment are also shown. After 24 h (SK-BR-3) or 48 h (MCF-7) of incubation with tested taxane (100 nM for SK-BR-3 cells and 300 nM for MCF-7 cells), levels of cleaved caspases were determined using western blot analysis and relevant antibodies (see “Materials and Methods”). Actin levels were used to confirm equal protein loading. The data shown were obtained in one representative experiment of two independent experiments.

SK-BR-3 cells with suppressed expression of caspase-2 (for the efficiency see previous section) were incubated with paclitaxel or SB-T-1216 (100nM) for 24 h. Subsequent western blot analysis showed significantly decreased cleavage of caspase-9 and caspase-3 due to inhibition of caspase-2 expression. No considerable change in the level of cleaved caspase-8 was observed. Surprisingly, the level of cleaved caspase-7 significantly increased (Figure 5).

MCF-7 cells (without functional caspase-3) with inhibited expression of caspase-2 were incubated with tested taxanes (300nM) for 48 h. Western blot analysis showed significantly decreased levels of cleaved caspase-8 and cleaved caspase-9. Decreased level of cleaved caspase-7 was not as pronounced (Figure 5).

### Effect of taxanes on cellular distribution of caspase-2

The effect of tested taxanes on cellular distribution of caspase-2 was assessed using confocal microscopy (see “Materials and Methods”).

**Figure 6:**
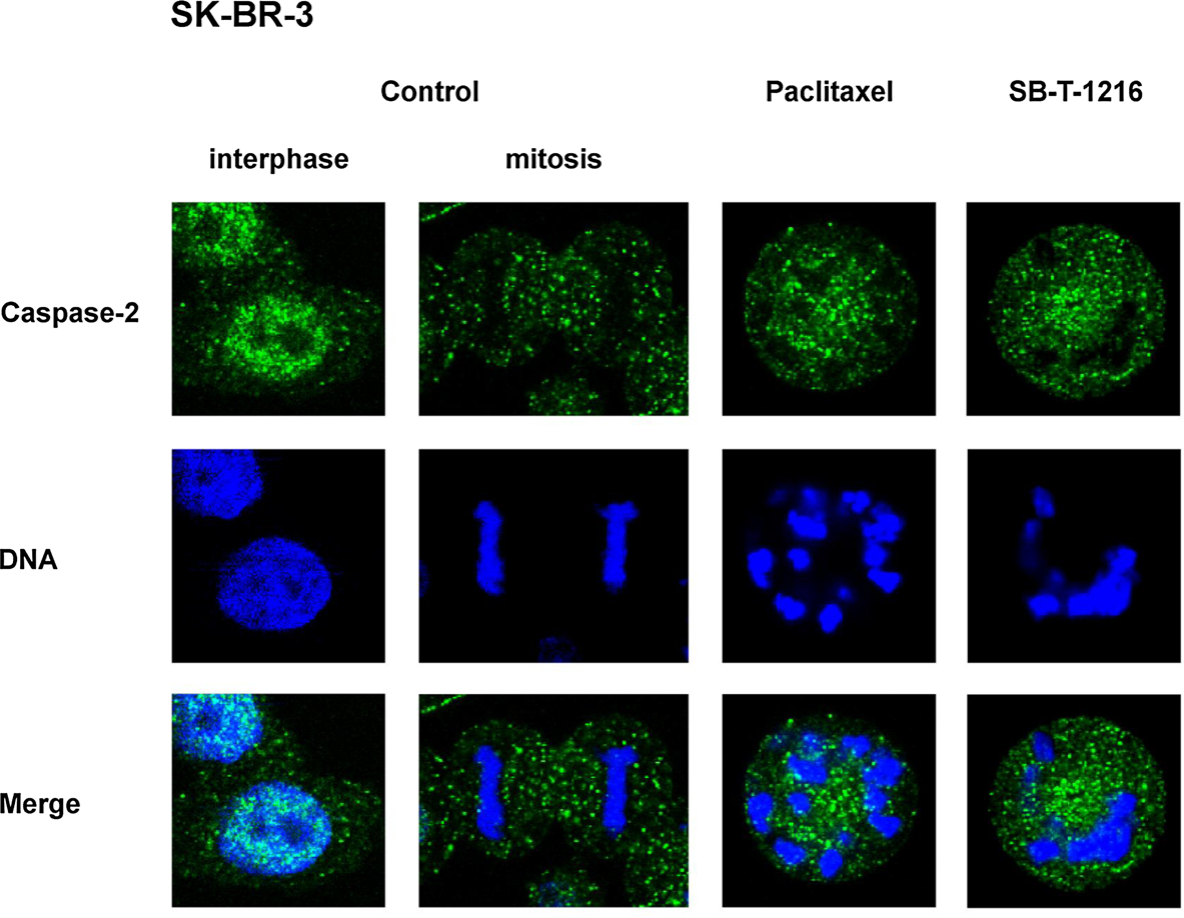
**Effect of paclitaxel and SB-T-1216 on cellular distribution of caspase-2 in SK-BR-3 cells.** Control cells were incubated without taxane. After 36 h of incubation with tested taxane (100 nM), the localization of caspase-2 and DNA position were detected using confocal microscopy, following staining with DAPI (DNA) and indirect immunofluorescence staining (caspase-2) with antibody against human caspase-2 (see “Materials and Methods”). The localization of caspase-2 (green), of DNA (blue) and the merge of caspase-2 and DNA within the cell are shown. The data shown were obtained in one representative experiment of two independent experiments.

Confocal microscopy showed that caspase-2 (detected by two different antibodies) seemed to be mainly found in the nucleus of control SK-BR-3 cells. However, caspase-2 did not colocalize with DNA, as we demonstrated when comparing interphase and mitotic control cells. After 36 h of incubation with paclitaxel or SB-T-1216 at death-inducing concentrations (100 nM), caspase-2 was released from the nucleus to the cytosol. This was probably due to the mitotic block following taxane application which is associated with the disintegration of the nuclear envelope (Figure 6). However, caspase-2 was not redistributed into mitochondria (data not shown). Similar data were obtained with MCF-7 cells (data not shown).

### Effect of taxanes on p53 activation

We assessed the activation of p53 by induction of p21^WAF1/CIP1^ expression employing real-time PCR (mRNA level) and western blot analysis (protein level).

**Figure 7:**
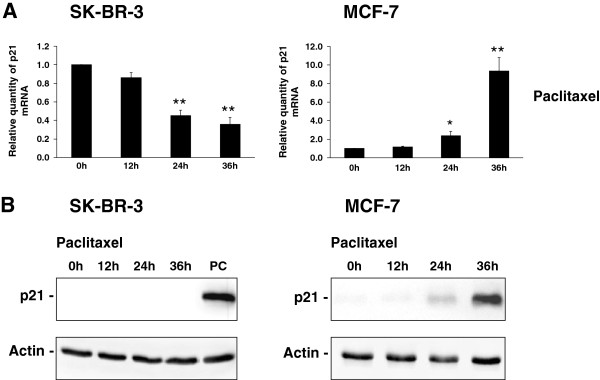
**Effect of paclitaxel on the level of (A) p21^WAF1/CIP1^ mRNA and (B) p21^WAF1/CIP1^ protein in SK-BR-3 and MCF-7 cells.** After 0, 12, 24 and 36 h of incubation with paclitaxel (100 nM for SK-BR-3 cells and 300 nM for MCF-7 cells), the level of mRNA was determined using RT-PCR and relevant primers and the level of protein was determined using western blot analysis and relevant antibodies (see “Materials and Methods”). Each column of mRNA data represents the mean of 4 experimental values ± SEM. *P < 0.05, **P < 0.01 when comparing the effect with that at 0 h. Actin levels of protein data were used to confirm equal protein loading. In the case of SK-BR-3 cells, MCF-7 cells after 36 h of incubation with paclitaxel were used as a positive control (PC). The data shown were obtained in one representative experiment of two independent experiments.

Within 36-h incubation of SK-BR-3 cells (nonfunctional p53) with paclitaxel at the death-inducing concentration (100 nM), the level of p21^WAF1/CIP1^ mRNA decreased to 40% of the original value (statistically significant decrease). Western blot analysis did not detect any p21^WAF1/CIP1^ protein in these cells during 36 h of incubation with the taxane (Figure 7). Similar data were obtained with SB-T-1216 in SK-BR-3 cells (data not shown).

With regard to MCF-7 cells (functional p53), p21^WAF1/CIP1^ mRNA level increased approximately 9-fold during 36 h of incubation with paclitaxel at the death-inducing concentration (300 nM). This pronounced and statistically significant increase of p21^WAF1/CIP1^ mRNA level corresponded to a pronounced increase in p21^WAF1/CIP1^ protein level (Figure 7). Again, similar data were obtained with SB-T-1216 in these cells (data not shown).

### Effect of taxanes on PIDD expression

Effect on PIDD expression was assessed using real-time PCR (mRNA level) and western blot analysis (protein level).

**Figure 8:**
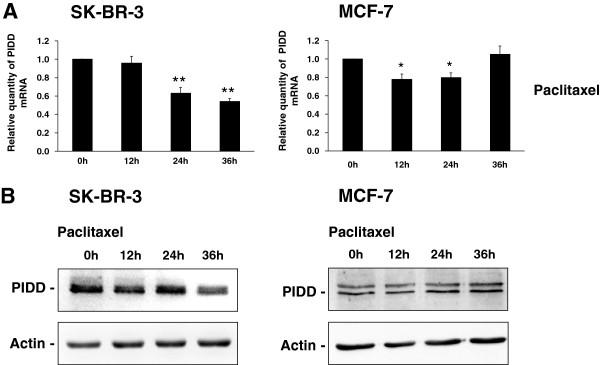
**Effect of paclitaxel on the level of (A) PIDD mRNA and (B) PIDD protein in SK-BR-3 and MCF-7 cells.** After 0, 12, 24 and 36 h of incubation with paclitaxel (100 nM for SK-BR-3 cells and 300 nM for MCF-7 cells), the level of mRNA was determined using RT-PCR and relevant primers and the level of protein was determined using western blot analysis and relevant antibodies (see “Materials and Methods”). Each column of mRNA data represents the mean of 4 experimental values ± SEM. *P < 0.05, **P < 0.01 when comparing the effect with that at 0 h. Actin levels of protein data were used to confirm equal protein loading. The data shown were obtained in one representative experiment of three independent experiments.

The level of PIDD mRNA showed a decrease (to 60% of the original value) during 36-h incubation of SK-BR-3 cells with paclitaxel at the death-inducing concentration (100 nM). PIDD protein level also seemed to decrease slightly after 36 h of incubation with paclitaxel (Figure 8). Similar data were obtained with SB-T-1216 (data not shown).

PIDD mRNA in MCF-7 cells showed similar levels during 36-h incubation with paclitaxel at the death-inducing concentration (300 nM). No significant change was found for PIDD protein level (Figure 8). As was the case for SK-BR-3 cells, similar data were obtained with SB-T-1216 (data not shown).

Similar data were obtained, when PIDD protein levels were assessed using flow cytometric analysis after staining with relevant primary and secondary antibodies, for both cell lines (SK-BR-3 and MCF-7) and both taxanes (paclitaxel and SB-T-1216) (data not shown).

### Effect of taxanes on the coimmunoprecipitation of caspase-2 and PIDD with RAIDD

Coimmunoprecipitation (see “Materials and Methods”) of RAIDD protein with other components of PIDDosome, i.e. PIDD protein and caspase-2, was assessed using subsequent western blot anylysis.

**Figure 9:**
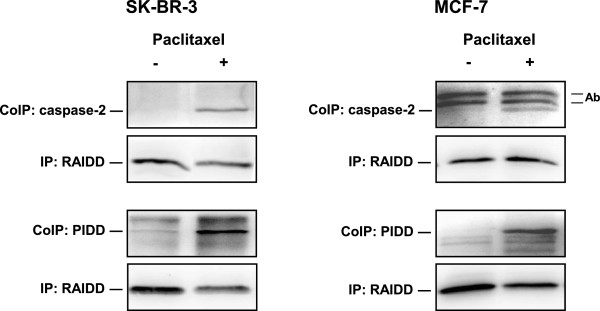
**Effect of paclitaxel on coimmunoprecipitation of caspase-2 and PIDD with RAIDD in SK-BR-3 and MCF-7 cells.** After 24 h of incubation with paclitaxel (100 nM for SK-BR-3 cells and 300 nM for MCF-7 cells), the level of precipitated RAIDD and coimmunoprecipitated caspase-2 as well as PIDD (detected by antibody against C-form) were determined using western blot analysis and relevant antibodies (see “Materials and Methods”). The heavy chain of the antibody used for RAIDD precipitation (Ab) is seen. The data shown were obtained in two independent experiments.

Both caspase-2 and PIDD protein (detected by antibody against C-form) were coimmunoprecipitated with RAIDD protein after 24-h incubation of SK-BR-3 cells with paclitaxel at the death-inducing concentration (100nM) (Figure 9).

Similarly, caspase-2 and PIDD protein were detected in coimmunoprecipitated complex with RAIDD protein in MCF-7 cells after 24 h incubation with paclitaxel at the death-inducing concentration (300 nM). Thus it seems that PIDDosome is formed in both SK-BR-3 and MCF-7 cells when taxane is applied at death-inducing concentrations.

### Effect of the inhibiton of RAIDD expression on taxane induced cell death

Employing RNA interference (see “Materials and Methods”), we assessed the effect of specific inhibition of RAIDD expression on cell death induction after taxane application.

**Figure 10:**
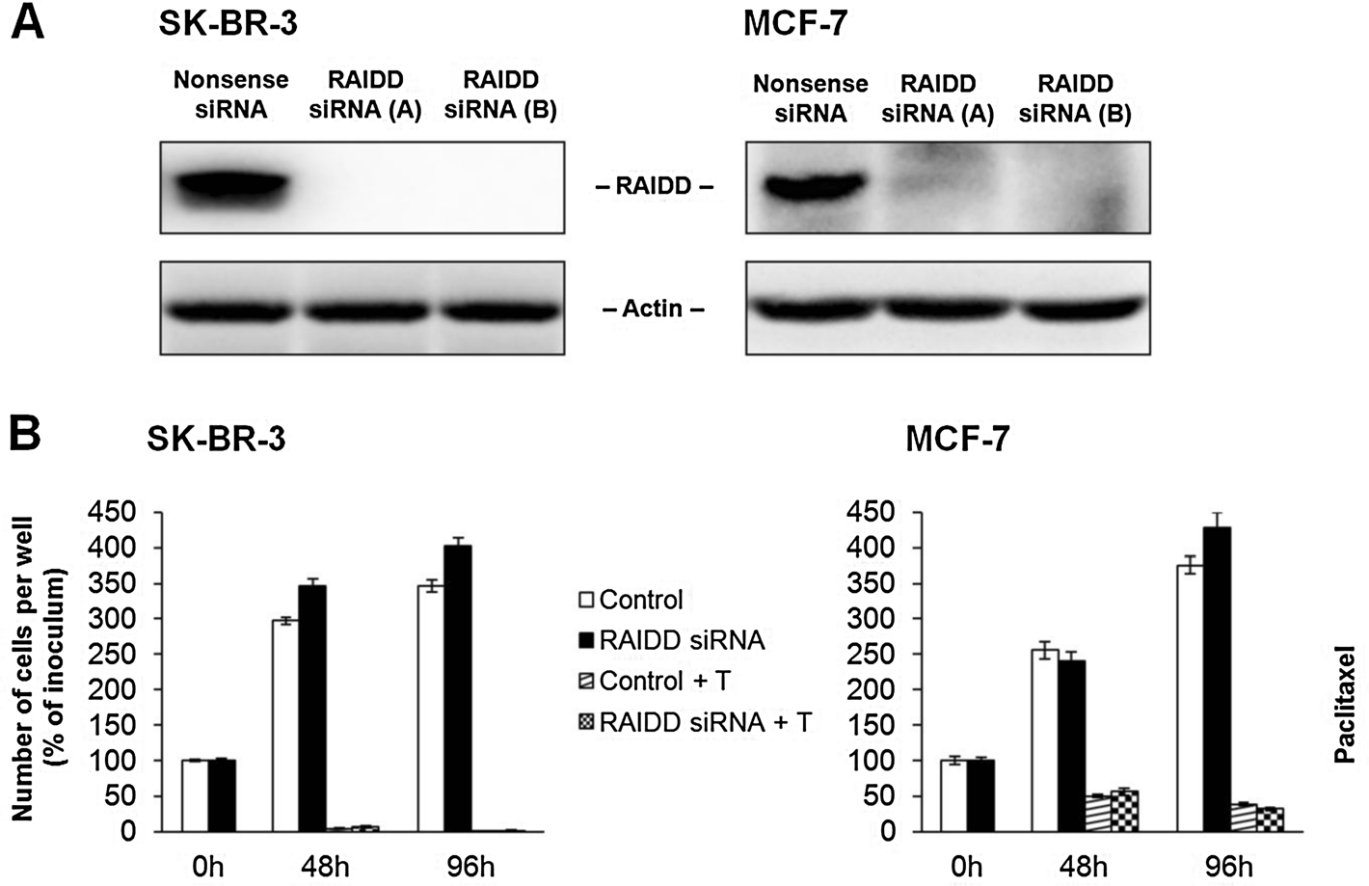
**Effect of the inhibition of RAIDD expression on the growth and survival of SK-BR-3 and MCF-7 cells after paclitaxel treatment.** (**A**) Efficiency of RAIDD suppression by two employed specific siRNA, i.e. **A** and **B**, in SK-BR-3 and MCF-7 cells is shown. Levels of RAIDD were determined using western blot analysis and relevant antibodies (see “Materials and Methods”). Actin levels were used to confirm equal protein loading. (**B**) The effect of specific RAIDD siRNA on the growth and survival of SK-BR-3 and MCF-7 cells after paclitaxel (T) treatment (100 nM for SK-BR-3 cells and 300 nM for MCF-7 cells) is presented. The cells were seeded at 7 × 10^3^ cells/200 μl of medium per well and prepared as described (see “Materials and Methods”). After 0, 48 and 96 h of incubation, the number of living cells was determined (see “Materials and Methods”). Each column represents the mean of 2 separate cultures ± SEM.

The inhibition of RAIDD expression by RNA interference was found to be highly efficient for both SK-BR-3 and MCF-7 cells (Figure 10A). Specific RAIDD siRNA did not significantly affect cell growth or survival in ether cell line (data not shown).

After 48 h as well as 96 h of incubation with paclitaxel at death-inducing concentration (100 nM), the inhibition of RAIDD expression did not result in any significant change in the number of surviving SK-BR-3 cells (Figure 10B).

Similarly for MCF-7 cells, the inhibition of RAIDD expression did not result in any significant change in the number of surviving cells after 48 h and 96 h of incubation with paclitaxel at death-inducing concentration (300 nM) (Figure 10B).

## Discussion

In our previous studies [7,14], we demonstrated that caspase-2 was significantly activated (up to 20-fold) along with other caspases (caspase-3, caspase-9 and caspase-8) during apoptosis induction by taxanes in some cancer cells. We have also shown that, at least in some cases, the mitochondrial pathway does not represent the main pathway of apoptosis induction by taxanes. Recently, we found that apoptosis was induced by taxane application in SK-BR-3 and MCF-7 breast cancer cells and that caspase-2 was also significantly activated in these cells. Furthermore, MCF-7 cells are without functional caspase-3, as we confirmed (data not shown). Thus it is reasonable to assume that caspase-2 could play an important role in apoptosis induction by taxanes in breast cancer cells.

In order to study the role of caspase-2 in apoptosis induction by taxanes in breast cancer cells, we employed a convenient model using SK-BR-3 and MCF-7 cells. SK-BR-3 cells have nonfunctional p53 and functional caspase-3 [39]. On the other hand, MCF-7 cells have functional p53, but they do not have functional caspase-3 [40]. The use of these two cell lines, with their opposite properties, could help to elucidate the role of caspase-2 in apoptosis induction by taxanes. It could particularly contribute to our knowledge concerning the relationship between caspase-2 activation and the activation of other caspases as well as the mechanism of caspase-2 activation itself.

We tested both a classical taxane paclitaxel and a novel (second-generation) taxane SB-T-1216. In our previous study with MDA-MB-435 and NCI/ADR-RES cell lines [14], SB-T-1216 was shown to be more effective than paclitaxel, particularly in NCI/ADR-RES cells resistant to paclitaxel. However, SB-T-1216 and paclitaxel seemed to use the same or similar mechanism of cell death induction [7,14]. In this study, MCF-7 cells were shown to be a slightly more resistant to taxanes than SK-BR-3 cells but both cell lines showed similar sensitivity to paclitaxel and SB-T-1216 (see Figure 1). Furthermore, it seems that paclitaxel and SB-T-1216 use the same mechanism of apoptosis induction (see Figures 2, 3, 4, 5, 6 and “Results”).

Significant activation of caspase-2 in SK-BR-3 and MCF-7 cells after taxane application at death inducing concentrations (see Figure 2) was confirmed using several different methods (see Figure 3). It is known that the measurement of caspase-2 activity can be affected by the activity of caspase-3 [41]. Thus, the measured activity of caspase-2 (see Figure 2) can be in fact a sum of caspase-2 and caspase-3 activities. It can explain the discrepancy in timing of measured caspase-2 activity (see Figure 2) and detected caspase-2 activation (see Figure 3). In MCF-7 cells, we only detected cleavage of procaspase-2 (see Figure 3A) under different conditions than procaspase-2 was detected. However, the problem of detecting the cleaved form of caspase-2 in MCF-7 cells could be a specific feature of these cells as described previously [42]. With regard to other cell types, several studies have demonstrated caspase-2 activation in various types of cancer cells after apoptosis induction by taxanes [37,38,43].

Several studies with human prostate cancer cells, human melanoma cells and mouse embryonic fibroblasts have shown that caspase-2 is required for apoptosis induction by taxanes. These studies employed various techniques using siRNA, caspase-2^-/-^ cells and specific caspase-2 inhibitors [21,23,44]. We also demonstrated that caspase-2 is required, at least partially, for apoptosis induction by taxanes in both studied breast cancer cell lines. The inhibition of caspase-2 expression using siRNA technique resulted in a significantly increased number of surviving cells following application of taxanes at death-inducing concentrations. MCF-7 cells, with inhibited caspase-2 expression, grew even after application of death-inducing concentrations of taxanes (see Figure 4). This effect could not be attributed to the stimulatory effect of siRNA application on cell proliferation (see Figure 4B).

In spite of the fact that several studies have shown the involvement of caspase 2 in apoptosis induction by various stimuli in breast cancer cells [27,34,35], the mechanism of caspase-2 involvement in apoptosis induction is not fully understood. Caspase-2 has been described as an apical caspase [21,23] as well as a possible executioner caspase [45], in various types of cancer cells, together with its functions which are independent of apoptosis [46,47]. In this study with SK-BR-3 and MCF-7 cells we demonstrated that, together with caspase-2, other caspases (caspase-8, caspase-9, caspase-3, caspase-7) were activated after application of taxanes.

The activation of caspase-9, -3 and -7 was significantly affected in SK-BR-3 cells with inhibited caspase-2 expression after cell death induction by taxane application. While the cleavage of caspase-9 and caspase-3 significantly decreased, caspase-7 cleavage increased. In MCF-7 cells (without functional caspase-3) the cleavage of caspase-9 was nearly blocked and the cleavage of caspase-7 was significantly decreased. Cleavage of caspase-8 decreased slightly in SK-BR-3 cells and significantly in MCF-7 cells (see Figure 5). The substantial inhibitory effect of the inhibition of caspase-2 expression on the activation of caspase-8 and caspase-9, as well as the activation of executioner caspase-3 in SK-BR-3 cells and executioner caspase-7 in MCF-7 cells, supports a suggestion that caspase-2 functions as an apical caspase. The increase of caspase-7 activation together with the decrease of caspase-3 activation, due to the inhibition of caspase-2 expression, in SK-BR-3 cells seems rather obscure (see Figure 5). Some kind of compensatory mechanism could be involved.

Additional information concerning caspase-2 function could come from testing the cleavage of caspase-2 substrates. However, there are only a few known specific substrates of caspase-2 such as golgin-160 [31,46]. In this study, we demonstrated golgin-160 cleavage after application of death-inducing taxane concentrations in both studied cell lines (see Figure 3B). Unfortunately, it was not very helpful in elucidating caspase-2 function without data connecting golgin-160 cleavage to other relevant events.

Caspase-2 has been found in the cytosol, Golgi complex, mitochondria and also in the nucleus of cells [46]. Regarding SK-BR-3 and MCF-7 cells, we showed that caspase-2 seemed to be primary localized in the nucleus. However, caspase-2 did not colocalize with DNA, as demonstrated with mitotic cells. Treating the cells with death-inducing taxane concentrations seemed to lead to a redistribution of caspase-2 from the nucleus to the cytosol (see Figure 6). Caspase-2 was probably released from the nucleus because of the long-term disintegration of the nuclear envelope associated with the mitotic block after application of taxanes. The question, whether caspase-2 is activated within the nucleus or in the cytoplasm, has not been answered yet [47]. In our case, long-term exposure of most of the cellular caspase-2 to the cytoplasmic environment, which can comprise caspase-2-activating capacity, could lead to caspase-2 activation. Thus, there could be a very simple explanation for caspase-2 activation in breast cancer cells after taxane application.

Caspase-2 activation is usually connected with PIDDosome formation and PIDD protein upregulation via the induction of expression by activated p53 [23-25]. We found significant p53 activation assessed by the induction of p21^WAF1/CIP1^ expression, after the application of death-inducing taxane concentration in MCF-7 cells with functional p53 [48]. On the other hand, in SK-BR-3 cells without functional p53 [39] we confirmed that there was no p53 activity (see Figure 7). In the next step, we assessed the effect of taxanes on PIDD expression. No significant effect of taxanes on PIDD upregulation was found in either SK-BR-3 or MCF-7 cells (see Figure 8). This means that p53 activation, and the subsequent PIDD upregulation, is not involved in caspase-2 activation.

Nevertheless, we detected some coimmunoprecipitation of RAIDD protein with both PIDD protein and caspase-2 in both cell lines 24 h after taxane application at the death-inducing concentrations. However, suprisingly the inhibition of RAIDD expression using siRNA technique did not affect the number of surviving SK-BR-3 as well as MCF-7 cells after taxane application at all. Therefore, we suggest that PIDDosome formation [49] does not represent the main platform for caspase-2 activation in breast cancer cells when apoptosis is induced by taxanes. Recently, other pathways of caspase-2 activation which circumvent PIDDosome formation have been reported [50], e.g. the activation of caspase-2 in DISC complex [51]. Therefore other pathways of caspase-2 activation in cells treated with taxanes should be considered.

## Conclusions

We can summarize that significant caspase-2 activation is associated with apoptosis induction by taxanes in tested breast cancer cells and that caspase-2 is required, at least partially, for the induction as well. Caspase-2 could be activated due its release from the nucleus and subsequent long-term exposure to the cytoplasmic environment after taxane application resulting in long-term disintegration of the nuclear envelope. Concerning the mechanism of caspase-2 activation, caspase-2 seems to be activated via other mechanism than PIDDosome formation. The activation of both initiator and executioner caspases after taxane application depends on caspase-2 expression. Thus we can suggest that caspase-2 functions as an apical caspase in apoptosis induction by taxanes.

## Materials and methods

### Materials

Paclitaxel was obtained from Sigma-Aldrich (St. Louis, MO, USA). SB-T-1216 [13] was synthesized at the Institute of Chemical Biology and Drug Discovery (Stony Brook, NY, USA). Taxanes were dissolved in DMSO (tissue culture quality) to obtain a 1 mM stock solution.

For western blot analysis, the following primary antibodies were used: mouse monoclonal antibody against caspase-3 (#9668), rabbit antibody against cleaved caspase-3 (#9661), rabbit antibody against cleaved caspase-7 (#9491), mouse monoclonal antibody against caspase-8 (#9746), and rabbit antibody against cleaved caspase-9 (#9505) from Cell Signaling Technology (Danvers, MA, USA), rat monoclonal antibody against caspase-2 (ALX-804-355-C100) and mouse monoclonal antibody against PIDD (ALX-804-837) from Enzo Life Science (Farmingdale, NY, USA), rabbit monoclonal antibody against caspase-2 (ab32021), rabbit polyclonal antibody against caspase-2 (ab 18737), goat polyclonal antibody against golgin-160 (anti-GOLGA3, ab40837), and rabbit monoclonal antibody against RAIDD (Ab52621) from Abcam (Cambridge, UK), rabbit polyclonal antibody against caspase-2 (H-145, sc-15379), mouse monoclonal antibody against p21 (F-5, sc-6246), goat polyclonal antibody against PIDD (S-17, sc-32161), and rabbit polyclonal antibody against PIDD (H-300, sc-2354) from Santa Cruz Biotechnology (Santa Cruz, CA, USA), and mouse monoclonal antibody against actin (AC-40, A3853) from Sigma-Aldrich.

For precipitation, Protein A/G PLUS- agarose beads (sc-2003) from Santa Cruz Biotechnology were used.

### Cells and culture conditions

Human breast carcinoma cell lines SK-BR-3 and MCF-7 were obtained from American Type Culture Collection (ATCC) (Rockville, MD, USA) and National Cancer Institute (Frederick, MD, USA). The cells were maintained in a culture medium at 37°C in a humidified atmosphere of 5% CO_2_ in air. The culture medium consisted of basic medium supplemented with 10% heat-inactivated fetal bovine serum (Biochrom AG, Berlin, Germany). The basic medium was RPMI 1640 medium (Sigma-Aldrich, St. Louis, MO, USA) containing extra L-glutamine (300 μg/ml), sodium pyruvate (110 μg/ml), HEPES (15 mM), penicillin (100 U/ml) and streptomycin (100 μg/ml), as previously described [52].

### Assessment of cell growth and survival

Cells were harvested and seeded at 20 × 10^3^ cells/100 μl of culture medium into the wells of a 96-well plastic plate. After 24-h preincubation period allowing cells to attach, the culture medium was replaced by either the culture medium without taxane (control) or with one of tested taxanes (paclitaxel or SB-T-1216) at desired concentrations. Cell growth and survival were evaluated after 96 h of incubation. The number of living cells was determined using a hemocytometer after staining with trypan blue [53].

### Measurement of caspase-2 activation

A commercial CaspGLOW^™^ Active Caspase Staining Kit (Biovision, Mountain View, CA, USA) was used to detect the active form of caspase-2, as previously described [54]. Cells (approximately 3 × 10^5^ cells per sample) were seeded and after a 24-h preincubation period allowing cells to attach, the culture medium was replaced by either taxane-free culture medium (control) or with medium containing taxane (paclitaxel or SB-T-1216) at desired concentrations. After the required incubation period, the cells were harvested using low-speed centrifugation and staining was performed according to the manufacturer’s instructions. Fluorescence was measured using a FACS Calibur cytometer (Becton Dickinson, San Jose, CA, USA).

### Real-time PCR

Cells were harvested and seeded at 1.2 × 10^6^ cells/6 ml of the culture medium into Petri dishes. After a 24-h preincubation period, the culture medium was either replaced by taxane-free culture medium (control) or with medium containing taxane (paclitaxel or SB-T-1216) at desired concentrations. Total RNA was isolated from SK-BR-3 and MCF-7 cells using a RNAeasy MiniKit (Qiagen, Hilden, Germany) according to the manufacturer’s instructions after the required incubation period.

Prepared RNA was reverse transcribed using a TaqMan Reverse Transcription Reagents kit (Applied Biosystems, Foster City, CA, USA) with random primers, in accordance with the manufacturer’s instructions. Transcribed cDNA was subjected to real-time quantitative PCR in an ABI Prism 7000 Sequence Detection System (Applied Biosystems) using a commercially available TaqMan Gene Expression Master Mix kit (Applied Biosystems) with TaqMan Gene Expression Assays (Applied Biosystems) for CDKN1A (cyclin-dependent kinase inhibitor 1A, p21), PIDD (p53-induced death domain protein), CASP2 (caspase-2) and for GAPDH (glyceraldehyde 3-phosphate dehydrogenase) as the control gene. All data were normalized relative to the amount of GAPDH cDNA in the sample and the 2^−ΔΔCt^ method was used to calculate relative changes in genes expression using ABI Prism 7000 SDS Software Version 1.1 (Applied Biosystems).

### Western blot analysis

Cells (approximately 1 × 10^7^ cells per sample) were seeded and taxanes were applied after 24-h preincubation. Cells were harvested after the incubation period by low-speed centrifugation, washed in PBS and centrifuged. Cell pellets were stored at -80°C. Frozen pelets were resuspended in RIPA buffer (Sigma Aldrich, St. Louis, MO, USA) containing a mixture of protease inhibitors (Sigma Aldrich). The protein lysate was centrifuged (14,000 rpm, 20 min, 4°C) and the supernatant was stored at -20°C.

Western blot was carried out with some modifications as has been previously described in detail [4]. Proteins separated by SDS-PAGE were blotted onto 0.2 μm nitrocellulose membrane PROTRAN BA 83, (Whatman-Schleicher and Schuell, Maidstone, UK) for 3 h at 0.25 A, using a MiniProtean II blotting apparatus (Bio-Rad, Hercules, CA). The membrane was blocked with 5% nonfat milk or 5% BSA in TBS for 15 min. TBS containing Tween-20 (0.1%) was used for washing. The washed membrane was incubated with the primary antibody. Following incubation (overnight, 4°C), the membrane was washed (three times) and then incubated for 1–2 h with the corresponding horseradish peroxidase-conjugated secondary antibody (Santa Cruz Biotechnology, Santa Cruz, CA, USA). Afterward, the membrane was washed three times and the chemiluminescence signal was detected using a Supersignal West Pico Chemiluminescence Substrate from Pierce (Thermo Fisher Scientific Inc., Rockford, IL, USA) and and the KODAK Gel Logic 1500 Imaging System (Eastman Kodak Company, Rochester, NY, USA).

### RNA interference

In order to optimize the RNA interference procedure, two independent siRNAs targeting the caspase-2 mRNA sequence, i.e. MISSION® esiRNA human CASP2 (Sigma Aldrich, St. Louis, MO, USA) and CASP2 siRNA (s2412, Applied Biosystems, Foster City, CA, USA), and two independent siRNA targeting the RAIDD mRNA sequence, i.e. two CRADD (RAIDD) Silencer® Select siRNAs (s16654 and s225028, Applied Biosystems, Foster City, CA, USA), were tested. Two different transfection agents, i. e. INTERFERin™ (Polyplus transfection™, Illkirch, France) and siPORT™ NeoFX™ Transfection Agent (Applied Biosystems) were also tested. We used GAPDH siRNA (Applied Biosystems) as a positive transfection control and Silencer® Negative Control siRNA (Applied Biosystems) as the non-targeting siRNA. The efficiency of caspase-2 and RAIDD inhibition was tested after 48-h incubation of cells with a medium containing transfection mixture for mRNA levels (real-time PCR, data not shown) and after 72-h incubation for protein levels (western blot analysis, Figure 4A). The efficiency of caspase-2 and RAIDD inhibition reached similar levels using any combination of the two tested transfection agents and the two siRNAs. For experiments, INTERFERin™ transfection agent (Polyplus transfection™), CASP2 siRNA (Applied Biosystems) and CRADD (RAIDD) siRNA s225028 (Applied Biosystems) were used.

Based on the manufacturer’s instructions (INTERFERin™ in vitro siRNA Transfection Protocol, Polyplus transfection™) we performed RNA interference in SK-BR-3 and MCF-7 cells. The cells were seeded at 7 × 10^3^ cells/200 μl of culture medium into a 96-well plate or at 7 × 10^4^ cells/2 ml of culture medium into a12-well plate for 24-h preincubation. The siRNA was diluted in OPTI-MEM® I Reduced Serum Medium (Gibco, Invitrogen™ Life Technologies, Carlsbad, CA, USA) for a final concentration of 5 nM siRNA. INTERFERin^™^ transfection agent (0.75 μl per 96-well and 4 μl per 12-well) was added. The mixture was incubated for 10 min at room temperature to form the transfection complex. Preincubation medium in wells was replaced by fresh culture medium (150 μl in 96-well plates and 1 ml in 12-well plates). Prepared transfection complex was added to fresh culture medium in cultivation wells and gently mixed. Cells were incubated with the medium containing transfection complex for 72 h. After incubation, the medium containing transfection complex was replaced with the culture medium containing tested taxane at the death inducing concentration for further analyses.

### Confocal microscopy

Cells were seeded onto coverslips (approximately 2 × 10^5^ cells per coverslip) and taxanes were applied after 24 h of preincubation as described above (see “Measurement of caspase-2 activation”). After 36 h of incubation for SK-BR-3 and 60 h of incubation for MCF-7, cells were fixed with 4% paraformaldehyde for 15 min at 37°C and permeabilized with 0.1% Triton X-100 in 4% paraformaldehyde for 15 min. After washing with PBS, cells were blocked with Image-iT™ FX signal enhancer (Molecular Probes, Invitrogen, Eugene, OR, USA) for 30 min. Next, cells were washed with PBS and stained with 30 μl of the primary antibody against caspase-2 (H-145, Santa Cruz Biotechnology, Santa Cruz, CA, USA or ab18737, Abcam, Cambridge, UK), diluted 1:50 in PBS, at 4°C overnight. Cells were than washed with PBS and incubated with 30 μl of Alexa Fluor® 488 goat anti-rabbit secondary antibody (Molecular Probes), diluted 1:200 in PBS, for 1 hour in a dark at room temperature. Finally, cells were washed again with PBS. Stained cells on coverslips were transferred onto a droplet of Vectashield^®^ Mounting Medium with DAPI (Vector Laboratories, Burlingame, CA, USA) and sealed. Samples were analyzed using a Leica TCS SP5 confocal microscope (Bannockburn, IL, USA) using a 63× oil objective at relevant excitation and emission wavelengths.

### Immunoprecipitation

First, 25 μl of agarose beads coated with bacterial proteins A and G (sc-2003) from Santa Cruz Biotechnology (Santa Cruz, CA, USA) were washed (resuspended in 0.5 ml of lysis buffer and centrifuged at 5000 rpm, 1 min) four times and finally resuspended in 0.5 ml of lysis buffer. 3.3 μl of anti-RAIDD (see “Materials”) antibody was added to the beads and mixture was incubated for 4 h in a refrigerator. Beads with bound antibody were washed as described and supernatant was removed. Beads with bound antibody were stored at 4°C.

Cell were harvested (see “Western blot analysis”) and lysed by non-denaturing lysis buffer (1% NP-40, 20 mM TRIS pH 7.4, 1 mM EDTA, 5% glycerol, 250 mM NaCl). Cell lysates were incubated for 20 min on ice and then centrifuged (14, 000 rpm, 15 min.) at 4°C. Supernatants containing cell proteins were stored on ice and protein concentrations were assessed.

Beads with bound antibody were diluted (to final volume of 100 μl) in lysate buffer containing 300 μg of cell proteins. The mixture was incubated overnight while agitated. After incubation, the mixture was centrifuged (6500 rpm, 7 min) and supernatant was discarded. Beads with bound immunocomplexes were washed twice as described with lysis buffer and twice with 50 mM Tris HCl pH 7.5. Washed beads with bound immunocomplexes were finally resuspended in 40 μl of sample buffer (see “Western blot analysis”) and heated for 15 min at 75°C to disintegrate the beads and release the immunocomplexes. 15 μl of the samples were loaded on 15% polyacrylamide gel for Western blot analysis (see “Western blot analysis”).

### Statistical analysis

Statistical significance of difference was determined using the Student’s *t*-test. P < 0.05 and P < 0.01 were considered statistically significant at the 5% and 1% levels, respectively.

## Competing interests

The authors declare that they have no competing interests.

## Authors’ contributions

MJ carried out western blot experiments and worked on the manuscript, KB carried out real-time PCR experiments and siRNA techniques, DK participated in western blot experiments and in coimmunoprecipitation, VNF carried out confocal microscopy, JŠ participated in coimmunoprecipitation, JF carried out flow cytometry analysis of caspases-2 activity, IZ and IO participated in the preparation of SB-T-1216 taxane, JK coordinated the experiments and helped to complete the manuscript. All authors read and approved the final manuscript.
